# Molecular docking analysis of a dermatan sulfate tetra-saccharide to human alpha-L-iduronidase

**DOI:** 10.6026/973206300191116

**Published:** 2023-12-31

**Authors:** Darinka P Durán-Gutiérrez, Marisol López-Hidalgo, Iliana M Peña-Gomar, Absalom Zamorano-Carrillo, Mónica L Gómez-Esquivel, José L Castrejón-Flores, César A Reyes-López

**Affiliations:** 1 Sección de Estudios de Posgrado e Investigación, ENMyH, Instituto Politécnico Nacional, Guillermo Massieu Helguera, No. 239, Fracc. "La Escalera", Ticomán, C.P. 07320, Mexico City, Mexico; 2 Departamento de Genética, Hospital IMSS-Bienestar Cuajimalpa, Cuajimalpa de Morelos, Mexico City, Mexico; 3 Instituto Politécnico Nacional, Unidad Profesional Interdisciplinaria de Biotecnología, Gustavo A. Madero, Mexico City, Mexico

**Keywords:** Molecular docking, structures, dermatan sulfate tetrasaccharide, human alpha-L-iduronidase, IDUA, GAG, MPS I

## Abstract

Human alpha-L-iduronidase (IDUA) is a 653 amino acid protein involved in the sequential degradation of glycos-amino-glycans (GAG), heparan sulfate (HS), and dermatan sulfate (DS). Some variants in the IDUA gene produce a deficient enzyme that causes un-degraded DS and HS to accumulate in multiple tissues, leading to an organ dysfunction known as muco-poly-saccharidosis type I (MPS I). Molecular and catalytic activity assays of new or rare variants of IDUA do not predict the phenotype that a patient will develop. Therefore, it is of interest to describe the molecular docking analysis, to locate binding regions of DS to IDUA to better understand the effect of a variant on MPS I development. The results presented herein demonstrate the presence of a polar/acidic catalytic site and a basic region in the putative binding site of DS to IDUA. Further, synthetic substrate docking with the enzyme could help in the predictions of the MPS I phenotype.

## Background:

Human alfa-L-iduronidase (IDUA) is a lysosomal glycoside hydrolase involved in the sequential degradation of glycosaminoglycans (GAG), heparan sulfate (HS), and dermatan sulfate (DS). IDUA is synthesized in the endoplasmic reticulum as a 653 amino acid precursor polypeptide and a 626-residue mature form, which folds into a TIM-barrel domain containing the catalytic site, an N-terminal beta-sandwich-folded domain and an immunoglobulin-like domain, also called Type III Fibronectin-like domain. This enzyme possesses six N-linked glycosylation sites that attach high-mannose-type and complex glycans [1,2]. Functionally, IDUA hydrolyzes the glycosidic bond of the L-iduronic acid (IdoA) residue from the non-reducing ends of DS and HS [1]. DS and HS are GAGs composed of repeating disaccharide units of glucuronic acid (GlcA) or iduronic acid (IdoA) and an amino sugar, N-acetylated glucosamine (GlcNac) or N-sulfated (GlcNS) or N-acetylgalactosamine (GalNac). The DS contains IdoA as the major uronic acid (UA) and a hexosamine that can be sulfated at 4-O, 6-O, 4-O, and 6-O disulfated or not sulfated [3]. While in HS, the dominant UA is the D-glucuronic acid that alternates with (1,4)-linked glucosamine (GlcN) residues, usually N-acetylated (GlcNAc), N-sulfated (GlcNS), or O-sulfated at C3 and/or C6 [4]. These GAGs are found in different tissues such as the cornea, sclera, blood vessel walls, heart valves, and umbilical cord, in the case of DS, and within the cell surface and in the extracellular matrix, in the case of HS [5].

Deficiency of IDUA enzymatic activity causes undegraded DS and HS to accumulate in multiple tissues, leading to organ dysfunction known as mucopolysaccharidosis type I (MPS I) [6,7]. More than 200 variants in the IDUA gene have been reported associated with the development of MPS I [8], generating a spectrum of symptoms that varies from severe for Hurler Syndrome (OMIM#607014), intermediate for Hurler-Scheie syndrome (OMIM#607015), and mild for Scheie syndrome (OMIM#67016). MPS I is clinically manifested by different signs and symptoms, such as skeletal dysplasia, shortening of the limbs, mental retardation, valvular heart disease, hearing loss, corneal opacity, hepatosplenomegaly, and umbilical and inguinal hernias. Untreated patients who develop most clinical signs early in life die from respiratory failure, heart disease, or brain damage within the first two decades of life [9]. The diagnosis of MPS I is supported by molecular and biochemical assays to confirm the association of the altered catalytic activity of IDUA with the clinical phenotype, for which enzymatic activity assays are performed with the synthetic substrate 4-methylumbelliferyl a-L-iduronide (4-MUI). To date, with molecular and enzymatic activity studies of rare or new variants of IDUA, it is not possible to predict the phenotype that a patient will develop. Hence, the search for tools that achieve this association is a challenge.

The most common pathogenic variants worldwide are W402X and Q70X, which introduce a premature stop codon and produce truncated inactive proteins, and P533R, a common missense variant observed in MPS I patients from different continents [10]. The crystallographic structure revealed that IdoA binds to IDUA through nine amino acid residues, including the catalytic residues Glu299 and Glu182. Furthermore, mannose 7 (Man7) attached to the high-mannose type glycan of the N-glycosylated residue Asn372 has been reported to establish polar and van der Waals contacts with the atoms of IdoA or its analogs that bind to the active site of IDUA. This mannose residue is located approximately 4.3 Å above the C5 carboxylate of IdoA analogs, suggesting that the enzyme uses this high-mannose chain to extend the back wall of the IdoA binding pocket upward, separating the bound IdoA from the solvent [1, 2]. Therefore, it is of interest to describe the possible interaction of IDUA with two natural substrate molecules derived from DS and a synthetic substrate is discussed, proposing a tool to predict the impact of some IDUA variants on the catalytic activity of the enzyme.

## Methods:

## Target structures:

The crystallographic structure of the human IDUA protein, overexpressed in Chinese hamster ovary cells from *Cricetulus griseus*, was obtained from the Protein Data Bank (PDB ID: 3w82, holo-form (IdoA bound to hIDUA); and 3w81, apo-form), both with a sequence length of 642 residues and 5 N-glycan structures. The atomic coordinates of the ligand, solvent, and heteroatoms were removed from the structure, preserving the reported glycosylation chains.

## Structures of ligand molecules:

The structures of a disaccharide and a tetra saccharide derived from DS were used in docking analyzes so as to evaluate the interaction of IDUA with natural substrates. The reported coordinates of a DS disaccharide were obtained from the PubChem Compound database (PubChem CID: 32756). The structure of the DS tetra saccharide was obtained using the CHARMM-GUI glycan reader and modeler with a glycan reader sequence format of BGALNA_4SUF-13A: AIDOA-14BGALNA_4SUF-13A: AIDOA. The designed structure was optimized using the Optimize Geometry tool of Avogadro software. The atomic coordinates of the synthetic substrate molecule, 4-methylumbelliferyl α-L-iduronide (4-MUI), were obtained from the bacterial ortholog of the human alpha-L-iduronidase structure deposited in PDB (PDB ID: 5NDX). Finally, the IdoA coordinates of the IDUA crystallographic structure were used as a control for the docking approach.

## Molecular dockings:

AutoDockTools4 [11] software was used in all docking experiments. The IDUA structure was kept as a rigid target molecule, while the ligands were tested as flexible molecules. Polar hydrogen atoms were added for IDUA using the AutoDockTools, the Kollman united partial charges were added for all atoms in the protein, and Gasteiger charges were assigned to the ligands. The position of iduronic acid in the IDUA crystallographic structure was used as a guide to center a 40 Å x 40 Å x 40 Å grid box with a spacing of 0.375 Å around the catalytic site. For docking experiments with the IdoA, the DS-derived disaccharide, and the synthetic substrate 4-MUI, a 46 Å x 52 Å x 40 Å (x, y, z) grid box was created with a spacing of 0.375 Å. For docking experiments with the tetra saccharide ligand, the grid box size was set to 50 Å x 58 Å x 40 Å (x, y, and z). The Lamarckian genetic algorithm was applied for the docking calculations. During the docking process, 10 different conformers were generated for each ligand with an initial population of 150 randomly placed individuals and a maximum number of 2.5 x 107 energy evaluations. The selection of the IDUA-bound ligand end position was ranked by lower binding energy and conservation of polar contacts with IdoA or disaccharide and tetra saccharide non-reducing end IdoA residues, compared to the crystallographic complex.

## Results:

**Figure 1:**
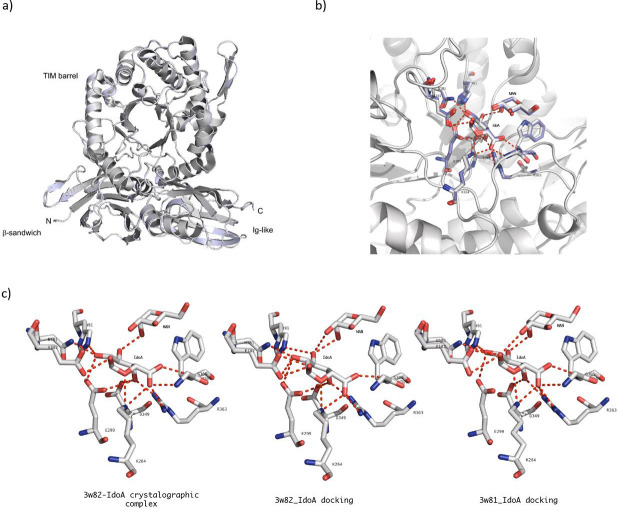
Crystallographic and molecular docking simulation complexes of IDUA-IdoA. a) Superposition of IDUA 3w81 (gray) and 3w82 (light blue) crystallographic structures. b) Close-up of the superposition of the IDUA catalytic site of the IdoA 3w81 docking complex (in gray) and the IdoA 3w82 crystallographic complex (in light blue). A stick representation of catalytic residues, binding residues, and IdoA molecules is shown. c) Polar contacts (red dotted lines) between catalytic and binding amino acids of IDUA and the IdoA in the 3w82 crystallographic complex (left), the 3w82 docking complex (center), and the 3w81 docking complex (right).

Molecular docking was carried out with the 3w81 (apo-structure) and 3w82 (holo-structure) IDUA crystallographic structures. A comparison of structures showed that no differences are appreciable when C-alpha atoms are aligned (RMSD 0.206 Å). The side chain of catalytic and binding residues presented the same position in both structures (Figure 1a,b), suggesting that in IDUA, the site of IdoA binding is structured independently of the presence of ligand. IDUA-IdoA docking experiments yielded complexes with IdoA coordinate close to the crystallographic complex (Figure 1b). In the IDUA-IdoA crystallographic complex, IdoA has polar contacts with nine IDUA residues and with Man7 of the N372-N-glycan (Table 1). The achieved complex of IdoA with the 3w82 structure obtained with Auto Dock reproduced native polar contacts with eight of the nine IDUA residues and the Man7 polar contact (Table 1). For the complex obtained with Auto Dock for structure 3w81 and IdoA, all polar contacts between the amino acid residues of IDUA and IdoA observed in the crystallographic complex of structure 3w82 were reproduced (Figure 1c and Table 1). The binding free energies of the crystallographic complex and those obtained with Auto Dock to the 3w81 and 3w82 structures were estimated with the PRODIGY-LIG program [12] in order to compare them. No significant differences were observed in this parameter for any complex, including the crystallographic one (Table 1). These results suggest that this methodological approach could predict possible sites of interaction between IDUA and its natural substrate.

Since the 3w81 IDUA crystallographic structure was resolved with a more improved resolution than the 3w82 structure (2.30 Å vs. 2.76 Å, respectively) and with more experimental information (97.2% completeness for range and 74,141 numbers of reflections for the 3w81 structure, compared to 95.7% completeness and 38,427 reflections for the 3w82 structure), resulting in a better overall IDUA experimental model, docking experiments for 4-MUI substrate and DS-derived disaccharide and tetra-saccharide molecules were carried out with the 3w81 structure.

Oligosaccharide structures generated using the CHARMM-GUI glycan reader was used as ligands to predict the potential binding site of DS-derived disaccharide and tetra-saccharide molecules. The ligands were given a reasonable level of degrees of freedom by allowing complete flexibility to their glycosidic torsion angles and hydroxyl groups. This approach allows comparisons between experimental and theoretical ligand structures, thus facilitating the estimation of the effect of ligand-induced fit on the outcome of the binding analysis [13].

**Figure 2:**
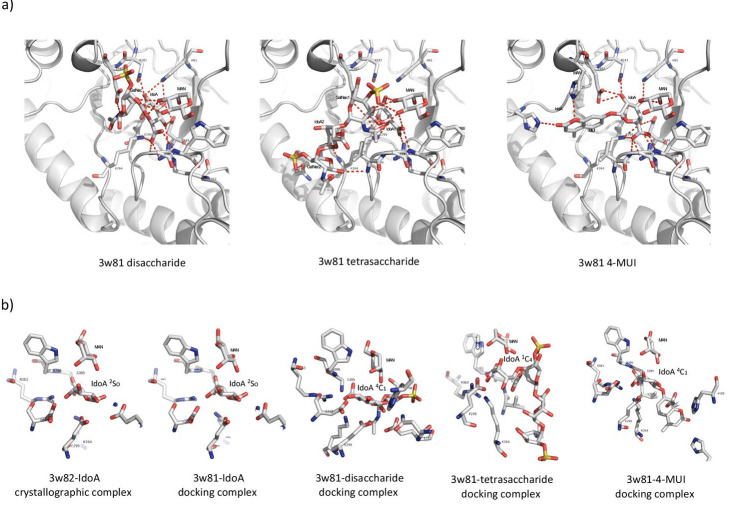
Molecular docking simulation complexes between IDUA and DS saccharides or the 4-MUI synthetic substrate. a) Polar contacts (red dotted lines) between catalytic and binding amino acids (stick representation) of IDUA and a disaccharide derived from DS (left), a tetrasaccharide (center), and the synthetic substrate 4-MUI (right). Carbon atoms are shown in gray, oxygen atoms in red, nitrogen atoms in blue, and sulfate atoms in yellow. b) Conformation of the IdoA residue observed in crystallographic and docking complexes. The conformation of each IdoA residue corresponds to the IUPAC nomenclature.

Docking experiments of IDUA with DS-derived disaccharide yielded a complex that positioned the IdoA residue of the ligand at the catalytic site of the IDUA, with atomic positions close to the IDUA-IdoA complex, conserving polar contacts with all residues associated with the binding and catalytic site reported from the crystallographic structure and observed with the IDUA-IdoA Auto Dock docking experiments. Additional polar contacts were observed through the Oe2 atom of Glu182, the GalNac N atom of the amino sugar residue, and the O3 and O4 atoms of Man7 with O3 and O14 of the same GalNac residue (Figure 2a). The -DG of this complex, estimated with PRODIGY-LIG, was slightly higher (about 1.8 kcal/mol) than that estimated for the IDUA-IdoA complex (Table 1).

The complexes obtained with a tetra-saccharide derived from DS showed that the contacts with the IdoA residue of the non-reducing end are conserved, except for the contacts with the atoms of the residues His91, Asn181, and Gly305 (Table 1 and Figure 2a). Furthermore, polar contacts were observed between O10 and the sulfate group of the first amino sugar residue of the tetra-saccharide with Man7. Polar contacts were also observed between the O5 of the second amino sugar residue of the tetra-saccharide and the N of Val304, as well as the O9 of the same amino sugar with the N of Lys264 and the Oe1 of Gln275 with the phosphate group of the same amino sugar residue. No polar contacts were observed with the second IdoA residue of the tetra-saccharide in any of the complexes obtained. It is important to highlight that although the IDUA residues that maintain polar contacts with the IdoA 1 and the GalNac of the tetra-saccharide are the same as those observed for the disaccharide, some of the atoms involved in these contacts differ (Table 1). The IdoA residue bound to the catalytic site for each complex acquires a different conformation. In the complex with IdoA, the conformation was 2SO; in the disaccharide, IdoA acquires a ^4^C_1_ conformation, and the IdoA residue at the non-reducing end of the tetra-saccharide presents a ^1^C_4_ conformation (Figure 2b). These different conformers can explain the difference in atoms that form the polar contacts in each complex but with the same residues of the IDUA catalytic site. In the docking carried out with the synthetic substrate 4-MUI, polar contacts were observed between the IdoA residue of the substrate and the same IDUA residues present in the crystallographic structure, maintaining the majority of contacts between the two molecules (Figure 2a). Furthermore, polar contacts were observed between the MUI group of the synthetic substrate and His185 and His226 of the IDUA (Table 1). In this same docking, it was observed that the IdoA residue of 4-MUI acquires a ^4^C_1_ conformation, similar to the conformation observed in the DS-derived disaccharide (Figure 2b).

## Discussion:

Prediction of residual enzymatic activity of IDUA variants is a major goal for early diagnosis and timely treatment of MPS I patients. Detailed molecular knowledge of how IDUA binds to its substrate can help estimate the effect of a variant on IDUA activity and predict a pathogenic effect in a carrier patient. The crystallographic structure of IDUA showed an IdoA binding site, which restricts the orientation of the GAG that can bind to the enzyme [1,2]. To our knowledge, there are no known structures from any organism that show the binding of IDUA to natural substrates. Some structures complexed with small sugar molecules derived from DS, which are not related to IDUA, have been reported. The crystallographic structure of a complex of DS with cathepsin K, a protease involved in the degradation of bone collagen that requires binding to GAG for adequate catalytic activity, showed that the binding site of DS is rich in Lys residues and Arg [14]. A less basic binding site, but with high polarity, has been reported in the interaction of bacterial chondroitin AC lyase and a DS-derived tetra-saccharide, where polar residues such as His, Asp, Asn, and Glu are relevant for binding and degradation of this GAG [15]. Interestingly, in human dermatan sulfate epimerase 1 (DS-epi1), an enzyme involved in the epimerization of position 5 of GlcA residues to form L-iduronic acid, but which does not hydrolyze this GAG, the active site and substrate-binding groove was found to shift from a negative to a positive surface potential as the pH decreases to the optimum pH of DS-epi1 and the pH found in the Golgi lumen [16].

**Figure 3:**
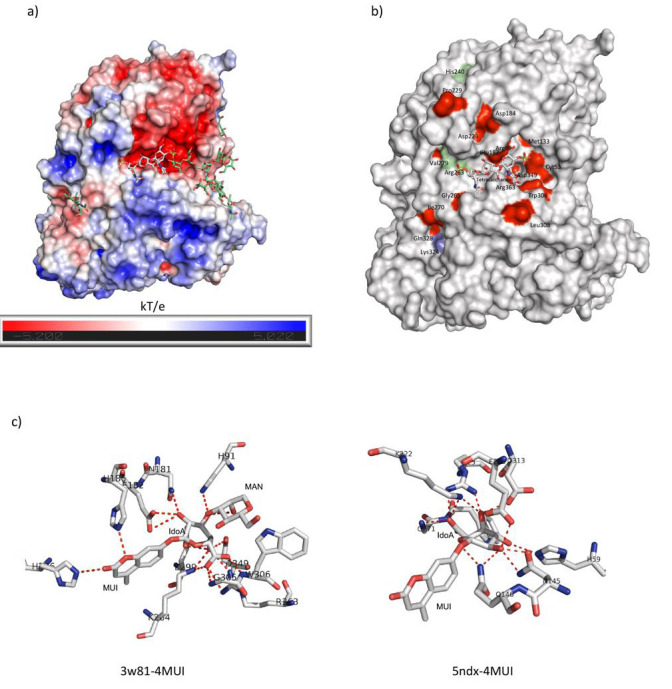
General characteristics of the binding site of IDUA and a tetra-saccharide derived from DS. a) Representation of the electrostatic potential of IDUA (calculated with APBS), visualized on the solvent-accessible surface colored from red to blue (from 5.2 kT/e to 5.02 kT/e) and simulation of molecular docking of a tetra-saccharide derived from DS. N-glycosylation of Asn415 and Asn372 are shown in green sticks and the tetra-saccharide molecule in gray sticks. b) Location of pathogenic variants of IDUA that do not incorporate an early stop codon and that generate severe (red surface), intermediate (light blue surface), or mild (green surface) MPS I phenotypes. c) Polar contacts of IDUA and 4-MUI from a molecular docking simulation (left) and the crystallographic structure of a bacterial ortholog of IDUA (5NDX) and 4-MUI (right).

These reports agree with the results presented in this work, which suggest that glycosaminoglycans could bind to IDUA through a groove formed in the barrel-like TIM domain between the glycosylation sites of Asn372 and Asn415, as predicted by the docking carried out with the tetra-saccharide. The amino acid residues that are part of the catalytic site and that have polar contacts with GAG of the DS type are more like the DS binding site reported for the bacterial chondroitin AC lyase, with a predominance of polar and/or acidic amino acid residues. The amino acid residues located toward Ans415 confer a more basic character to the surface of the protein, where the presence of Arg and Lys is greater, like the DS binding site in cathepsin K, where basic residues are abundant (Figure 3a). These observations suggest that the catalytic sites of the enzymes that degrade DS could have a more polar/acidic character compared to the sites that bind DS, but where no catalytic processes are carried out for the degradation of this GAG. The presence of variants along this basic binding site could modify the observed surface potential, which could alter the interaction of the substrate with IDUA. This idea is reinforced by the observation that pathogenic variants that did not introduce an early stop codon in IDUA have a high frequency of appearance in the proposed ligand-binding groove (Figure 3b). Importantly, the majority of mutations occurring in this region are associated with the Hurler phenotype, and only one is associated with the Scheie phenotype, highlighting the importance of this region in IDUA activity and reinforcing the idea that substrate binding could be there.

On the other hand, molecular docking showed us that the synthetic substrate 4-MUI binds similarly to IDUA, as does a DS-derived disaccharide, with a similar DG binding. Molecular docking suggests that 4-MUI could bind to pathogenic variants present outside the active site with binding energies like those observed for the wild-type IDUA structure. These findings could explain the previously reported lack of correlation between the results of in vitro enzymatic activity with 4-MUI and the phenotype observed in patients carrying some MPS I variants [17]. Interestingly, the crystallographic structure of an ortholog of human IDUA complexed with the synthetic substrate 4-MUI (PDB ID: 5NDX), a protein expressed in *Rhizobium leguminosarum* bv. *Trifolii* showed that the residues that participate in the binding to ligand are like those observed in the complexes obtained with the IDUA (Figure 3c). It is feasible that a larger synthetic substrate that binds to a broader region of the enzyme could sense changes in the groove where the glycosaminoglycan chain is binding, allowing the correlation of enzymatic activity with an MPS I phenotype.

## Conclusions:

The degradation of GAGs is a process in which various enzymes participate; one of them is IDUA. In humans, there are several variants that affect the catalytic capacity for IDUA to cleave the IdoA from the non-reducing end of the DS. The results of the molecular docking of IDUA with ligands derived from DS provide us with an approach to better understand the effect of a variant on the development of MPS I. These analyses allowed us to better understand the discrepancy in the predictions of catalytic activity evaluated with 4-MUI and the phenotype that some patients develop. Additional studies are necessary to evaluate the impact of distant variants on the structuring of the proposed DS binding site, as well as to assess their impact on the binding of natural and synthetic substrates.

## Figures and Tables

**Table 1:** Polar contacts and free energy of binding of IDUA complexes

**IDUA Residue**	**Crystallographic**	**Docking**				
	**3w82 IdoA**	**3w82 IdoA**	**3w81 IdoA**	**3w81- disaccharide**	**3w81 tetrasaccharide**	**3w81 4-MUI**
His91	N^ε2^-O2 (3.1Å)	N^ε2^-O3 (3.4Å)	N^ε2^-O2 (2.9Å)	N^ε2^-O3 (3.3Å)^1^		N^ε2^-O3 (3.2Å)^6^
				N^ε2^-O2 (3.5Å)^1^		
Asn181	N^δ2^-O2 (2.8Å)	N^δ2^-O2 (2.9Å)	N^δ2^-O2 (2.8Å)	Nδ^2^-O2 (2.7 A)		N^δ2^-O2 (2.9Å)^6^
Glu182	O^ε2^-O1 (2.9Å)	O^ε2^-O1 (2.9Å)	O^ε1^-O1 (2.9Å)	O^ε2^-N2 (2.9Å)^2^	O^ε2^-O2 (2.9Å)^3^	O^ε1^-O1 (2.7Å)^6^
		O^ε1^-O1 (3.0Å)	O^ε2^-O1 (3.5Å)			O^ε2^-O2 (3.3Å)^6^
		O^ε2^-O2 (3.1Å)				
His185					N^ε2^-O5 (3.5Å)^3^	N^ε2^-O8 (2.9Å)^7^
His226						N^ε2^-O9 (3.2Å)^7^
Lys264	N^ζ^-O5 (3.2Å)	N^ζ^-O5 (2.8Å)	N^ζ^-O5 (3.1Å)	N^ζ^-O6A (2.9Å)^1^	N^ζ^-O2 (3.3Å)^3^	N^ζ^-O4 (2.6Å)^6^
	N^ζ^-O6A (3.2Å)	N^ζ^-O6A (2.7Å)	N^ζ^-O6A (2.7Å)		N^ζ^-O4 (2.4Å)^3^	N^ζ^-O6A (2.8Å)^6^
					N-O6 (3.2Å)^5^	
Gln275					O^ε1^-OSA (3.2Å)^5^	
Glu299	O^ε2^-O2 (2.8Å)	O^ε2^-O2 (3.4Å)	Oε^2^-O2 (3.0Å)	O^ε2^-O2 (2.8Å)^1^	O^ε1^-O2 (2.5Å)^3^	O^ε1^-O4 (2.7Å)^6^
	O^ε1^-O4 (3.4Å)	O^ε1^-O4 (3.2Å)		O^ε1^-O3 (3.2Å)^1^	Oε^2^-O4 (2.5Å)^3^	
Val304					N-O4 (3.3Å)^5^	
Gly305	N-O6A (2.9Å)	N-O6A (3.1Å)	N-O6A (3.1Å)	N-O6A (2.7Å)1	N-O5 (3.9Å)^3,^*	N-O6A (3.0Å)^6^
Trp306	N-O6B (2.7Å)	N-O6B (2.7Å)	N-O6B (2.8Å)	N-O6B (2.9Å)1	N-O6B (2.9Å)^3^	N-O6B (2.6Å)^6^
Asp349	O^δ1^-O4 (3.1Å)	O^δ1^-O4 (3.1Å)	O^δ2^-O4 (2.7Å)	O^δ1^-O3 (3.0Å)^1^		O^δ1^-O4 (3.1Å)^6^
				O^δ1^-O4 (2.6Å)^1^		
Arg363	N^η1^-O6A (3.3Å)	N^η1^-O6A (2.9Å)	N^η1^-O6A (2.9Å)	N^η1^-O6A (2.9Å)^1^	N^η2^-O6A (2.6Å)^3^	N^η1^-O6A (3.2Å)^6^
	N^η2^-O4 (2.4Å)	N^η2^-O4 (2.6Å)	N^η2^-O6A (2.6Å)	Nη^2^-O4 (2.6 A)^1^		N^η2^-O4 (3.0Å)^6^
			N^η1^-O4 (2.7Å)			
Man	O2-O3 (2.7Å)	O2-O3 (3.0Å)	O2-O3 (2.8Å)	O3-O5 (2.8Å)^1^	O3-O3 (2.4Å)^3^	O2-O3 (3.3Å)^6^
		O3-O3 (3.3Å)	O3-O1 (3.3Å)	O3-O1 (3.0Å)^1^	O3-O1 (2.7Å)^3^	
				O4-O6 (3.4Å)^2^	O4-O4 (2.5Å)^4^	
					O4-OSC (3.3Å)^4^	
Binding affinity& (kcal/mol)	-6.96	-6.9	-6.95	-5.13	-7.89	-5.48
^1^Disaccharide IdoA;
^2^disaccharide GalNac;
^3^tetrasaccharide IdoA1;
^4^tetrasaccharide GalNac1;
^5^tetrasaccharide GalNac2;
^6^4-MUI IdoA;
^7^4-MUI methyl umbelliferyl;
*Distances larger than 3.5 Å; & Calculated by PRODIGY-LIG
